# Impaired Microcirculation and Vascular Hemodynamics in Relation to Macrocirculation in Patients With Systemic Lupus Erythematosus

**DOI:** 10.3389/fmed.2021.722758

**Published:** 2021-11-01

**Authors:** Christina Svensson, Per Eriksson, Niclas Bjarnegård, Hanna Jonasson, Tomas Strömberg, Christopher Sjöwall, Helene Zachrisson

**Affiliations:** ^1^Department of Clinical Physiology, University Hospital, Linköping, Sweden; ^2^Division of Diagnostics and Specialist Medicine, Department of Health, Medicine and Caring Sciences, Linköping University, Linköping, Sweden; ^3^Division of Inflammation and Infection, Department of Biomedical and Clinical Sciences, Linköping University, Linköping, Sweden; ^4^Department of Biomedical Engineering, Linköping University, Linköping, Sweden

**Keywords:** SLE, microcirculation, augmentation index (AIx), ultrasound, intimal medial thickness (IMT), microvascular dysfunction

## Abstract

**Introduction:** Systemic lupus erythematosus (SLE) is associated with premature cardiovascular disease (CVD) and mortality, unexplained by traditional risk factors. Impairment of microcirculation and vascular hemodynamics may represent early signs of vascular affection. We hypothesized that studies of microcirculation and pulse waves may provide additional information, compared to ultrasound (US) alone, for the detection of early vascular disease in SLE.

**Methods:** Sixty well-characterized SLE-patients (52 women, eight men; mean age 43.21 ± 1.3 years) characterized by lupus nephritis (LN; *n* = 20), antiphospholipid syndrome (APS; n = 20) or skin and joint involvement (*n* = 20) and 60 healthy controls were included. Microcirculatory peak oxygen saturation (OxyP) was evaluated using a novel combined laser Doppler flowmetry/diffuse reflectance spectroscopy method. Pulse waves were recorded in the radial artery by the aid of applanation tonometry in order to calculate central augmentation index (AIx75). Intima-media thickness (IMT) and plaque occurrence were evaluated using high frequency US, in carotid and central arteries.

**Results:** Lower OxyP (84 ± 8 vs. 87 ± 5 %, *p* = 0.01) and higher AIx75 (17.3 ± 13.9 vs. 10.0 ± 14.2 %, *p* = 0.005) were seen in the SLE cohort. OxyP was inversely correlated with IMT in internal carotid artery (ICA), (*R* = −0.32, *p* = 0.01). AIx75 correlated with IMT in common carotid artery (CCA), (*R* = 0.36, *p* = 0.005), common femoral artery (CFA), (*R* = 0.43, *p* = 0.001), and ICA (*R* = 0.27, *p* = 0.04). AIx75 correlated negatively with OxyP (*R* = −0.29, *p* = 0.02). SLE-patients with plaque had lower OxyP values (80 ± 8 vs. 85 ± 7 %, *p* < 0.001) and higher AIx75 (23.0 ± 11.6 vs. 15.5 ± 14.2 %, *p* < 0.001) compared to those without plaque.

**Conclusion:** Impaired microcirculation and vessel hemodynamics were observed in SLE. These methods correlated with IMT and plaque occurrence. The importance of early macro- and micro-circulatory vascular affection for increased risk of CVD in SLE will be followed-up in future studies.

## Introduction

Systemic lupus erythematosus (SLE) is a multi-organ autoimmune inflammatory disease primarily affecting young females (1). Patients with SLE have an increased risk of cardiovascular disease (CVD) with accelerated atherosclerosis and higher mortality rates compared to the general population (2, 3). In middle-aged female patients with SLE, the increased risk for coronary heart disease can be as high as 50-fold (4).

To evaluate the risk of CVD, ultrasound (US) with measurements of intima media thickness (IMT) constitutes a validated and established method to assess early atherosclerosis (5). Previous studies of cardiovascular risk in SLE, including IMT and plaque assessment, have focused mainly on common carotid artery (CCA), (3, 6, 7). In a previous study from our group, we found an increased number of plaques in SLE compared to age- and sex-matched healthy controls using high frequency US of multiple vessel areas. In addition, we observed increased wall thickness with predominantly medium echogenic appearance in several arterial areas, predominantly in the aortic arch (8). This appearance can be seen in several inflammatory diseases (9, 10), with increasing age, (11) or as an early sign of atherosclerosis (12). Compared to inflammatory vessel wall appearance, atherosclerosis presents with a more heterogeneous, irregular vessel wall thickness.

Arterial stiffness in large arteries is considered as a decisive factor for systolic pressure and is a predictor of cardiovascular events (13). Pulse wave analysis (PWA) presented as augmentation index (AIx) is a measure of the universal cardiovascular condition and is altered by changes in for instance peripheral vascular tone and arterial stiffness.

According to prior studies (14), an increase in AIx has predictive value for future cardiovascular events and mortality. In hypertension, monitoring of AIx for risk assessment is recommended, although aging is a contributing factor for arterial stiffness (15). Earlier studies of women with SLE have indicated increased stiffness of their elastic central arteries as measured with pulse wave velocity (PWV), (16, 17). This may be one factor contributing to the increased cardiovascular risk seen in this cohort.

Several tools to assess microcirculation have been used clinically, i.e., capillaroscopy, infrared thermography and different laser techniques measuring microcirculation perfusion (18). Studies with different types of laser-based measurements to investigate microcirculation perfusion in SLE have been performed, but studies are scarce (18–20).

For evaluation of microcirculation in the skin, we employed a novel fiber-optic system that combines laser Doppler flowmetry (LDF) and diffuse reflectance spectroscopy (DRS). The system estimates red blood cell tissue fraction, speed resolved perfusion and oxygen saturation (21, 22). Previous studies using this method have shown that the system can discriminate blood perfusion from different blood-flow speeds (23), which may enable measurement of healthy and dysfunctional microcirculatory flow. The system has also been used to study microcirculatory perfusion in patients with type-2 diabetes (24) and in the Swedish Cardiopulmonary bioImage Study (SCAPIS), a large population-based cohort of men and women aged 50–65 year (25).

The aim of this study was to assess vascular hemodynamics in relation to macrocirculation in patients with SLE. We hypothesized that microcirculation and pulse waves may provide additional information, compared to ultrasound (US) alone, for the detection of early vascular disease in SLE.

## Materials and Methods

### Subjects

The study population (Supplementary Table 1) has previously been described in detailed (8) and was part of a regional Swedish quality register (26). Patients above 63 years of age were excluded due to increased age-dependent background risk of atherosclerosis (27), whereas only patients ≥23 years of age were considered. In order to compose a balanced study population, the 60 patients were stratified into three phenotypic subgroups with different manifestations of SLE. The subgroups were matched between each other 1:1:1 according to sex and age; 20 cases met the renal disorder ACR criterion, i.e., lupus nephritis (LN) in the absence of antiphospholipid syndrome (APS); 20 cases met the APS criteria (28) in the absence of LN; and 20 cases had exclusively skin and joint involvement in the absence of LN and APS (8, 26).

Acquired organ damage was assessed by the Systemic Lupus International Collaborating Clinics (SLICC)/American College of Rheumatology (ACR) damage index (SDI) and disease activity by the SLE disease activity index 2000 (SLEDAI-2K) for each patient, recorded from their closest regular visit to rheumatologist (29, 30). Mean time between examination and disease activity assessment was 3.8 months. 50/60 cases (83%) were of Caucasian ancestry.

Sixty healthy age- and sex-matched (i.e., 1:1 to the 60 SLE cases), non-medicated (except for contraceptives) controls without clinical signs of inflammatory or atherosclerotic disease, were examined using the same protocol (US, microcirculation and vascular hemodynamics) as for the patients. The healthy controls were all of Caucasian ethnicity and hospital employees.

### Background Variables

Height, weight, waist circumference and sagittal abdominal diameter were measured in all subjects. Variables concerning age, sex, smoking habits, physical activity, and ongoing pharmacotherapy were collected by oral request and medical records. Physical activity was defined as activity with increased heart rate (occasions/week). Blood pressure was determined with oscillometric technique (Dinamap PRO 200 Monitor, Critikon, Tampa, FL, USA).

### Microcirculation

**Figure 1:**
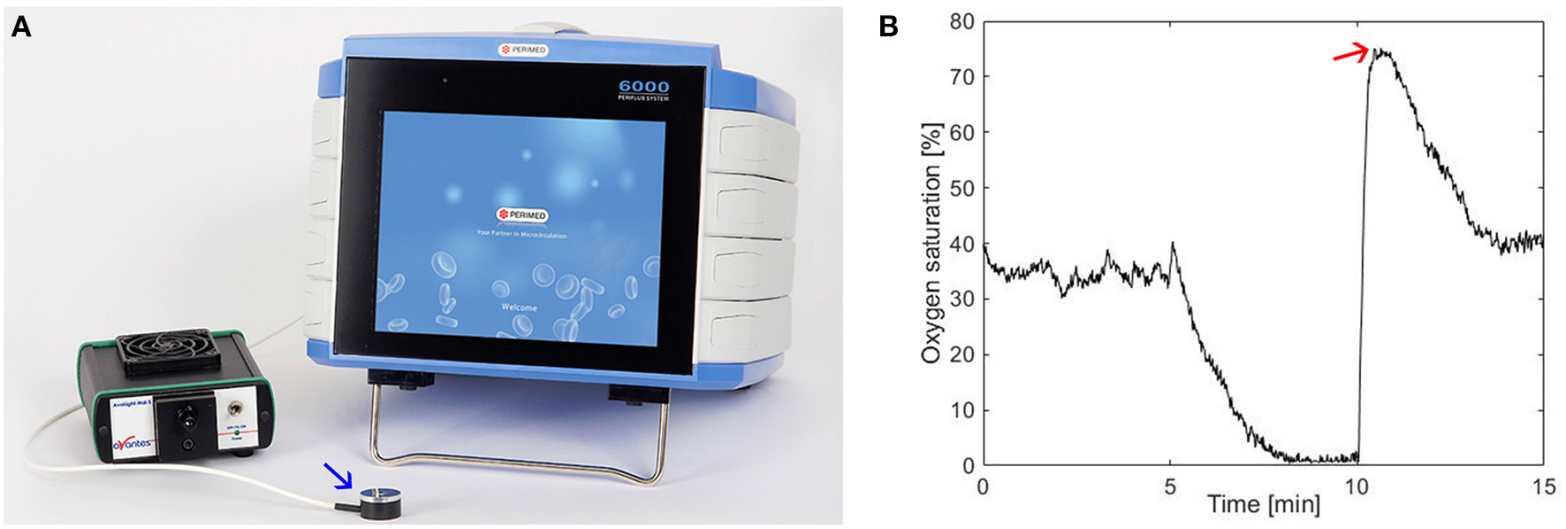
**(A)** The PeriFlux 6000 EPOS (Perimed) system with the probe (blue arrow) which was placed on the left forearm. **(B)** Oxygen saturation (%), baseline, during arterial occlusion (between 5 and 10 min), and in the post-ischemic. Red arrow denotes oxygen saturation peak.

Microcirculatory oxygen saturation was assessed with PeriFlux 6000 EPOS system (Enhanced Perfusion and Oxygen Saturation, Perimed, Järfälla, Sweden). A sphygmomanometer cuff was placed on the left upper arm, the probe (with both DRS and LDF) of the EPOS system (Figure 1A) was attached on the left forearm, ~10 cm below the cuff. A baseline measurement period of 5 min was followed by a 5-min suprasystolic occlusion of the upper arm ending with a 5-min post-ischemic measurement. Assessment of peak oxygen saturation was performed in the post-occlusive reactive hyperemia phase (PORH), (Figure 1B). PORH refers to the increase in blood flow that follows vascular occlusion involving endothelial vasodilatation. Based on the results of Jonasson et al., OxyP was selected as the most robust value to report (25, 31). This parameter reflects overall microcirculatory function associated with vasodilator capacity and is better than perfusion values to discriminate between diseased patients and healthy controls.

### Pulse Wave Analysis

**Figure 2:**
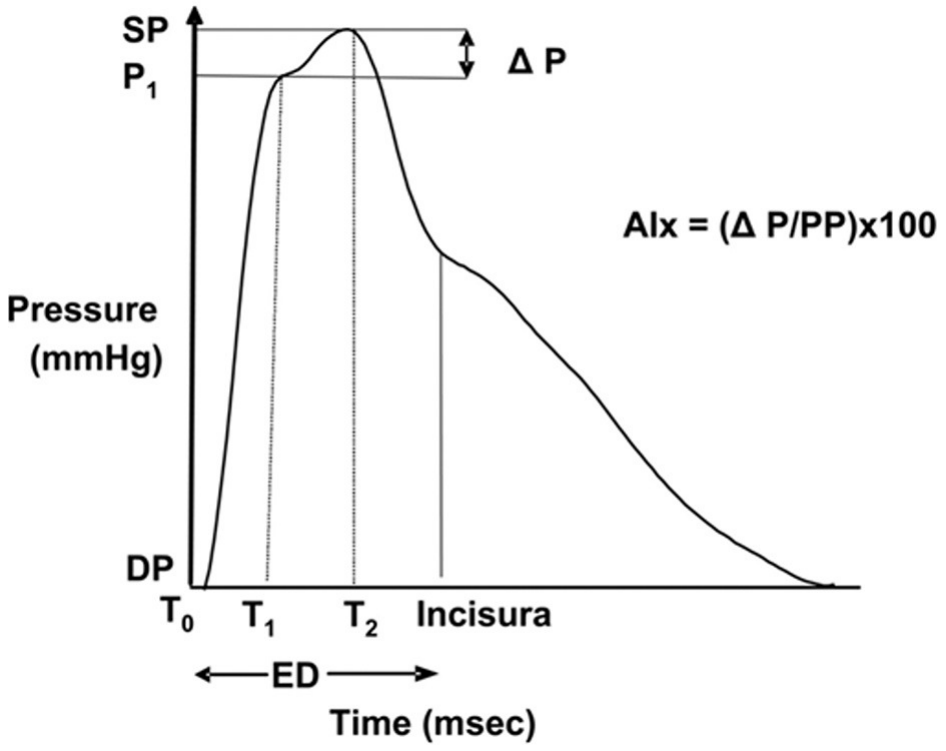
Pulse pressure waveform, T0 indicates the start of the waveform; T1, duration from start of waveform to the first peak (outgoing pressure wave); T2, duration from start of waveform to the second peak (reflected pressure wave); ED, ejection duration; SP, central aortic systolic pressure; DP, central aortic diastolic pressure; P1, height difference between the minimum pressure and the pressure at the first peak; ΔP, difference between maximal pressure (central aortic systolic pressure) and P1, pressure at the first peak; PP, pulse pressure; and AIx, augmentation index. Image with permission from AtCor Medical.

PWA was performed with applanation tonometry (SphygmoCor^®^ system, model MM3, AtCor Medical, Sydney, Australia), of the right radial artery. The locally recorded peripheral pressure waveform is traced by partly compressing the artery with an external high fidelity pressure probe (tonometer). Augmentation index adjusted to heart rate 75 (AIx75) was calculated. AIx is defined as [(Difference between the second and first systolic peak pressure)/Pulse Pressure] x100. This index denotes the relative aortic pulse pressure amplification in late systole from reflection waves (Figure 2). All measurements were made in at least duplicate, a mean of two reliable measurements (defined and calculated by SphygmoCor software as “operator index”) was calculated (32).

### Ultrasound

Ultrasound measurements were performed with a GE Logic E9 US system (LOGIQ E9 XDclear 2.0 General Electric Medical Systems US, Wauwatosa, WI, USA) with L2-9 MHz and C1-6 MHz transducers. IMT was measured in the common carotid artery (CCA), internal carotid artery (ICA), subclavian artery (SCA), axillar artery (AxA), common femoral artery (CFA), superficial femoral artery (SFA) and the aortic arch. Both sides were investigated. The procedure of IMT measurements has been described previously (8). The IMT cut-off value of ≥0.9 mm in CCA was chosen due to the latest ESH/ESC hypertension guidelines (33). For the aortic arch a higher cut-off value (≥1.2 mm) was chosen due to generally higher aortic arch IMT values among our healthy controls and according to results from earlier studies (34). Subjective vessel wall assessment regarding wall appearance and plaque occurrence was performed in carotid and central arteries as described earlier (8).

### Laboratory Measurements

As previously described (8), standard cardiovascular and inflammatory laboratory tests, including lipid profile, plasma creatinine and C-reactive protein with high-sensitive technique (hsCRP), anti-double-stranded DNA antibodies, and plasma complement protein 3 (C3) and 4 (C4), were controlled for at the closest regular visit to rheumatologist (35).

### Examination Procedure

The participant had to rest 15 min before the test, which was performed in a room with a temperature of 25°C, dim lighting and no outer disturbances. A standardized examination procedure was used in all individuals. The examination procedure started with US measurements followed by bilateral blood pressure measurements and pulse wave analysis of the right radial artery. The examination procedure ended with evaluation of microcirculation.

All participants were asked to refrain from coffee and nicotine use 4 h prior to the measurements.

The same vascular sonographer performed all physiologic examinations and the following off-line measurements. The sonographer was blinded to which subgroup of SLE the patients belonged, but not to whether the participants were patients or controls.

### Statistical Methods

AIx75, OxyP and IMT are presented as mean ± SD. Differences between the whole SLE group and controls were calculated using Student's *t*-test. χ^2^ test was performed for categorical variables. Differences between subgroups were calculated using one-way ANOVA with Bonferroni *post hoc* test. Pearson's correlation test, as well as univariate linear regression were used to test any relationship between AIx75 and OxyP and each of the variables in Table 1. Multivariate linear and logistic regression were used to examine factors explaining AIx75 and OxyP. All variables significant in the univariate model were combined and a stepwise procedure was performed eliminating non-significant (*p* ≥ 0.05) variables until a multiple model with only significant variables remained. For missing data, no imputation analysis was performed. Statistical analyses were performed using SPSS version 25.0 (IBM, Armonk, NY USA).

**Table 1:** Detailed characteristics of included patients and controls presented as mean ± SD or *n* (%).

	**All SLE (*n* = 60)**	**Controls (*n* = 60)**
	**Mean ± SD**	**Mean ± SD**
**Background variables**		
Age at examination (years)	43.2 ± 11.3	43.01 ± 11.4
Female gender, *n* (%)	52 (87)	52 (87)
Duration of SLE (years)	12.0 ± 9.4	N/A
SDI	0.8 ± 1.1	N/A
SLEDAI-2K	2.0 ± 2.1	N/A
Serologically active clinically quiescent SLE, *n* (%)	29 (48)	N/A
Lupus nephritis, *n*	20	N/A
APS, *n*	20	N/A
Skin & Joint, *n*	20	N/A
**Traditional risk factors and laboratory data**		
Body mass index (BMI) (kg/m^2^)	26.0 ± 4.2^**^	24.0 ± 3.3
Waist circumference (cm)	92.4 ± 12.1^***^	83.0 ± 10.0
Sagittal abdominal diameter (cm)	20.6 ± 3.9^**^	18.8 ± 2.7
Ever smoker (former or current), *n* (%)	14 (23) ^***^	0
Physical activity (times/week)	1.4 ± 1.6	1.8 ± 1.7
Systolic blood pressure (mm Hg)	115 ± 26	112 ± 18
Diastolic blood pressure (mm Hg)	73 ± 11^*^	68 ± 8
Diabetes mellitus, *n* (%)	1 (2)	0
Raynaud's phenomenon, *n* (%)	16 (27)	9 (15)
Estimated glomerular filtration rate (mL/min/1,73 m^2^)	84 ± 16	Not available
Total cholesterol (mmol/L)	4.7 ± 1.0	4.9 ± 1.1
High-density lipoprotein (HDL) (mmol/L)	1.6 ± 0.5	1.7 ± 0.4
Low-density lipoprotein (LDL) (mmol/L)	2.6 ± 0.8	2.6 ± 0.9
Triglycerides (TG) (mmol/L)	1.1 ± 0.7	1.2 ± 0.6
Anti-dsDNA (IU/mL)	86 ± 200	N/A
Complement protein 3 (g/L)	1.0 ± 0.2	N/A
Complement protein 4 (g/L)	0.2 ± 0.1	N/A
High-sensitivity CRP (mg/L)	2.2 ± 2.8	2.0 ± 3.7
**Medical treatment, ongoing**		
Antimalarial agents, *n* (%)	54 (90)	0
Antihypertensives, *n* (%)	20 (33)	0
Beta-blockers, *n* (%)	5 (8)	0
ARB/ACE inhibitors, *n* (%)	15 (25)	0
Other antihypertensives, *n* (%)	4 (7)	0
Glucocorticoid therapy *n* (%)	31 (52)	0
**Mean daily Prednisolone dose (mg)**	4.5	0
Warfarin therapy, *n* (%)	11 (18)	0
Antiplatelet therapy, *n* (%)	11 (18)	0
Statin therapy *n* (%)	5 (8)	0
DMARD therapy, *n* (%)	27 (45)	0
Mycophenolate mofetil, *n* (%)	16 (27)	0
Methotrexate, *n* (%)	5 (8)	0
Azathioprine, *n* (%)	3 (5)	0
Sirolimus, *n* (%)	2 (3)	0
Dehydroepiandrosterone, *n* (%)	1 (2)	0
Biologics, *n* (%)	4 (7)	0
Bortezomib, *n* (%)	1 (2)	0
Rituximab, *n* (%)	1 (2)	0
Belimumab, *n* (%)	2 (3)	0

*SLE patients compared to all controls*.

* *p <0.05*,

** *p <0.01*,

*** *p <0.001*.

*ACE, angiotensin converting enzyme; APS, Antiphospholipid syndrome; ARB, angiotensin II receptor blocker; CRP, C-reactive protein; DMARDs, Disease Modifying Anti-Rheumatic Drugs; LN, lupus nephritis; N/A, not applicable; SDI, SLICC/ACR damage index; SLE, Systemic lupus erythematosus*.

### Ethics Considerations

Oral and written informed consent was obtained from all patients and healthy controls. The study protocol was performed according to the Declaration of Helsinki and approved by the Regional Ethics Board in Linköping (ref. M75-08, 2013/33-31 and ref. 2017/572-32).

## Results

Basic demographics, laboratory data and ongoing medical therapies are shown in Table 1. Seventeen of the 60 patients (28%) had low-density lipoprotein (LDL) levels >3.0 mmol/L), but all patients on regular statin therapy (*n* = 5) showed LDL levels ≤2.7 mmol/L. No significant differences were seen between SLE and controls except for body mass index (BMI), waist circumference, sagittal abdominal diameter, diastolic blood pressure and smoking habits.

### Microcirculation and Pulse Wave Analysis

The average OxyP of the entire SLE group was significantly decreased compared to controls 84 ± 8 vs. 87 ± 5 % (*p* = 0.01). No significant differences with regard to the phenotypic SLE subgroups were found, although the LN group had the lowest values (82 ± 10 %). AIx75 values were increased in SLE patients compared to controls (17.3 ± 13.9 vs. 10.0 ± 14.2 %, *p* = 0.005). No significant differences with regard to the phenotypic subgroups were found, although the APS group showed the highest values (18.8 ± 15.2 %). When comparing gender, no significant differences were found for OxyP, whereas AIx75 differed significantly. AIx75 in females was 19.0 ± 13.7 vs. men 6.8 ± 10.7 % (*p* < 0.001). In controls, AIx75 for females was 11.6 ± 13.3 vs. men −0.6 ± 15.8 % (*p* = 0.02).

A significant inverse correlation was observed between OxyP and AIx75 (*R* = −0,29, *p* = 0.02).

### OxyP and AIx75 in Relation to IMT

OxyP was inversely correlated with IMT in ICA, (*R* = −0.32, *p* = 0.01). IMT in other vessel areas was not correlated with OxyP.

**Figure 3:**
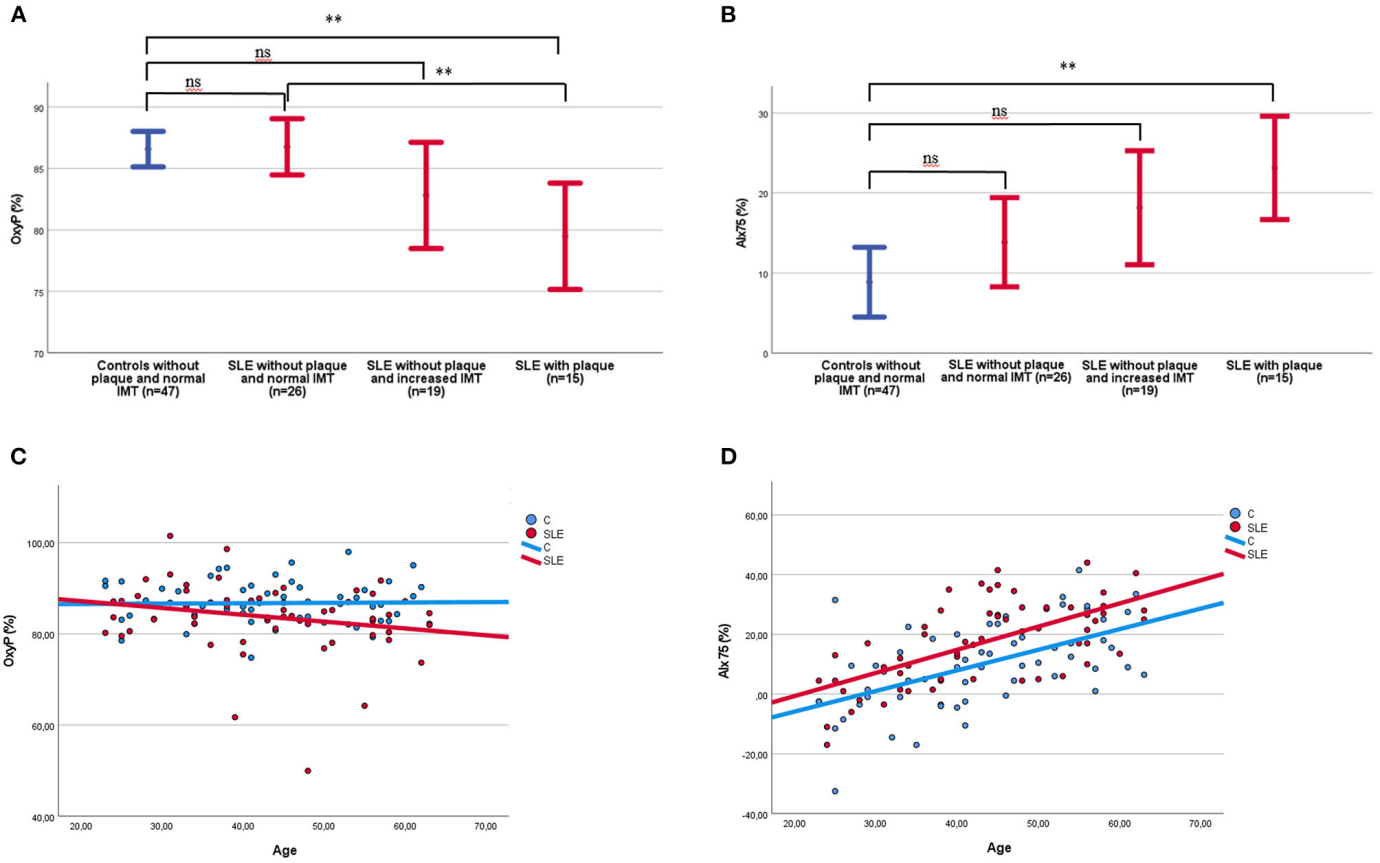
**(A)** OxyP (%) among patients and controls shown in relation to increased IMT in the aortic arch and plaque occurrence. Two controls with plaque were excluded in the figure. IMT, intima media thickness; OxyP, peak oxygen saturation; SLE, systemic lupus erythematosus. **(B)** AIx75 (%) among patients and controls shown in relation to increased IMT in the aortic arch and plaque occurrence. Two controls with plaque were excluded in the figure. IMT, intima media thickness; SLE, systemic lupus erythematosus. **(C)** OxyP among patients and controls shown in relation to age, SLE; *R* = −0.22 (ns), Controls; *R* = 0.02 (ns), **(D)** AIx75 (%) among patients and controls shown in relation to age. SLE *R* = 0.63 (*p* < 0.001); Controls *R* = 0.56 (*p* < 0.001). **p < 0.01.

We observed a difference in OxyP between (1) controls, (2) patients with normal IMT in the aortic arch without plaque, (3) patients with increased IMT in the aortic arch without plaque, and (4) patients with plaque (87 ± 5, 87 ± 6, 83 ± 9, 80 ± 8 %, respectively, *p* = 0.001). As demonstrated in Figure 3A, a significant difference was seen between controls and patients with plaque, and between patients with normal IMT in the aortic arch without plaque and patients with plaque.

AIx75 correlated with IMT in CCA (*R* = 0.36, *p* = 0.005), CFA (*R* = 0.43, *p* = 0.001) and ICA (*R* = 0.27, *p* = 0.04). Other vessel areas showed no significant correlation with AIx75.

AIx75 in the groups mentioned above were 8.9 ± 15.0, 13.9 ± 13.8, 18.2 ± 14.7, and 23.1 ± 11.7 %, respectively, (*p* = 0.04). AIx75 differ significantly between controls and patients with plaque (Figure 3B).

When comparing patients with or without plaque, regardless of normal or increased IMT in the aortic arch, the group with plaque had lower OxyP values (80 ± 8 vs. 85 ± 7 %, *p* < 0.001) and higher AIx75 (23.0 ± 11.6 vs. 15.5 ± 14.2 %, *p* < 0.001) compared to those without plaque.

### Relation of OxyP to Traditional and SLE Associated Risk Factors

Relation between OxyP and traditional and disease associated risk factors are shown in Table 2. In the univariate analysis of OxyP, waist circumference, sagittal abdominal diameter, presence of Raynaud's phenomenon, angiotensin II receptor blocker (ARB)/angiotensin-converting enzyme (ACE) inhibitor treatment, and statin therapy, were related to OxyP. However, when all significant variables were included in a multiple linear regression model, only the presence of Raynaud's phenomenon (*p* = 0.04) and statin therapy (*p* = 0.001) were negatively associated with OxyP (R = 0.51).

**Table 2:** OxyP and AIx75 related to background variables, traditional risk factors, laboratory tests and medical treatment in a univariate regression model among all 60 patients with SLE.

**All SLE (*****n*** **=** **60)**		**OxyP**			**AIx75**	
		**B**	***p*** **-value**		**B**	***p*** **-value**
**Background variables**						
Age at examiantion (years)		−0.150	NS		0.776	**<0.001**
Male gender		0.050	NS		−12.149	0.020
Duration of SLE (years)		−0.082	NS		0.529	0.005
SLICC/ACR damage index		0.105	NS		3.444	0.030
APS		1.139	NS		2.150	NS
LN		−2.315	NS		−1.075	NS
Skin & Joint		1.177	NS		−1.075	NS
**Traditional risk factors and laboratory tests**						
Body mass index (BMI) (kg/m^2^)		−0.274	NS		0.665	NS
Waist circumference (cm)		−0.632	0.010		0.987	0.030
Sagittal abdominal diameter (cm)		−0.175	0.040		0.404	0.006
Ever smoker (former or current)		−3.387	NS		0.905	NS
Physical activity (occasions/week)		1.141	NS		−2.375	0.034
Systolic blood pressure (mm Hg)		−0.044	NS		0.104	NS
Diastolic blood pressure (mm Hg)		−0.111	NS		0.774	**<0.001**
Raynaud's phenomenon		−5.811	0.010		5.415	NS
Estimated glomerular filtration rate (mL/min/1,73 m^2^)		0.039	NS		−0.347	0.002
Total cholesterol (mmol/L)		−0.640	NS		6.176	0.001
High-density lipoprotein (HDL) (mmol/L)		−0.201	NS		9.734	0.013
Low-density lipoprotein (LDL) (mmol/L)		−0.259	NS		3.567	NS
Triglycerides (TG) (mmol/L)		−1.828	NS		6.188	0.010
Anti-dsDNA (IU/mL)		−0.004	NS		−0.013	NS
Complement protein 3 (g/l)		−5.697	NS		0.354	0.006
Complement protein 4 (g/l)		−15.669	NS		0.262	0.040
High-sensitivity (hs) CRP (mg/L)		−0.021	NS		1.410	0.030
**Medical treatment, ongoing**						
Antimalarial agents		−4.899	NS		−0.176	NS
Antihypertensives		−2.138	NS		0.003	NS
Beta-blockers		−3.792	NS		13.700	0.030
ARB/ACE inhibitors		−6.492	0.005		2.567	NS
Other antihypertensives		−4.752	NS		6.732	NS
**Glucocorticoid therapy**		−3.519	0.080		1.896	NS
*Mean daily dose (mg)*		−0.712	0.070		−0.129	NS
Warfarin therapy		−2.033	NS		−1.420	NS
Antiplatelet therapy		0.176	NS		0.214	NS
**Statin therapy**		−12.727	**<0.001**		11.409	NS
**DMARD therapy**		−3.627	NS		−0.328	NS

*Red boxes denote variables remaining significant in multiple analysis. Bold p-values represent p < 0.001. OxyP, Peak oxygen saturation; AIx75, Augmentation Index correlated for heart frequency 75; ACE, angiotensin converting enzyme; APS, antiphospholipid syndrome; ARB, angiotensin II receptor- blocker; B, Beta coefficient; CRP, C-reactive protein; DMARD, Disease Modifying Anti-Rheumatic Drugs; LN, Lupus nephritis*.

OxyP was not associated with age (Figure 3C), duration of SLE, SDI, SLEDAI-2K, C3, C4 or anti-dsDNA levels.

Self-reported physical activity (occasions/week) were not associated with OxyP, neither in SLE nor in controls.

### Relation of AIx75 to Traditional and SLE Associated Risk Factors

Relation between AIx75 and traditional and disease associated risk factors are shown in Table 2. In the univariate analysis of AIx75, age, female sex, SLE duration, SDI, waist circumference, sagittal abdominal diameter, diastolic blood pressure, total cholesterol, high- density lipoprotein (HDL), triglycerids, C3/C4, hsCRP and beta-blocking therapy, correlated with AIx75. eGFR and physical activity correlated negatively with AIx75.

When all significant variables were included in a multiple linear regression model, age (Figure 3D), (*p* < 0.001), female sex (*p* < 0.001) and diastolic blood pressure (*p* < 0.001) remained as significant risk factors for AIx75 (*R* = 0.63).

There was a significant difference in AIx75 in relation to self-reported physical activity in SLE. For no physical activity AIx75 was 19.4 ± 13.1 % (*n* = 31), for physical activity 1–3 times/week 18.31 ± 2.5 % (*n* = 23), and for physical activity ≥4 times/week 3.1 ± 17.1 % (*n* = 6), (*p* = 0.02). In the controls, no significant differences were seen.

### Age Impairment on OxyP and AIx75 in SLE and Healthy Controls

**Figure 4:**
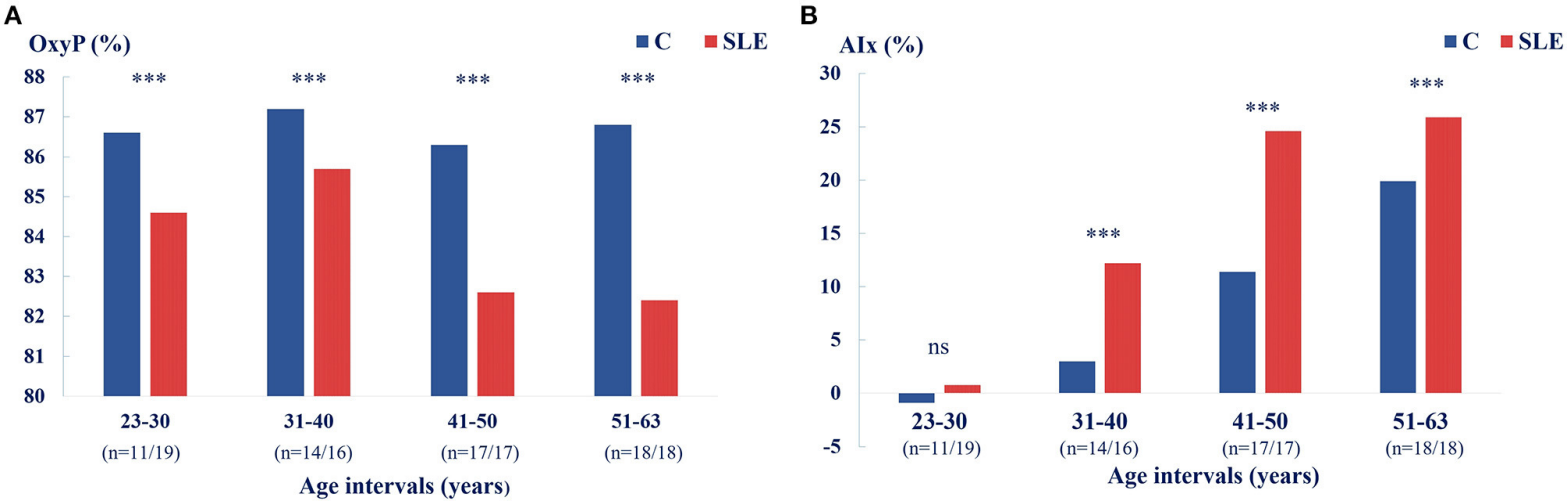
**(A)** Microcirculation reflected by OxyP (%) decreases with age in SLE patients but not in controls, *** *p* < 0.001. **(B)** AIx75 (%) among patients and controls shown in relation to age groups, *** *p* < 0.001.

As OxyP was lower, and AIx75 was higher, in SLE patients compared to controls, and as OxyP tended to decrease with age in SLE but not in controls (Figure 3C), we analyzed OxyP and AIx75 in different age groups (Figures 4A,B). In SLE, decreased OxyP levels were found in all age groups (*p* < 0.001). Increased AIx75 levels were found in three of four age groups (*p* < 0.001, ns in the youngest group).

## Discussion

This study of well-characterized SLE patients with clinically low disease activity showed impaired microcirculatory reactivity assessed by OxyP, and signs of premature arterial aging assessed by pulse wave recordings, when compared to healthy controls. Significant correlations with IMT in different vascular areas and plaque occurrence were also detected.

For assessment of microcirculation in SLE, prior studies have used different methods such as capillaroscopy, laser imaging techniques and infrared thermography (18).

In this study we used a novel fiber-optic method combining LDF/DRS for estimating oxygen saturation in the microcirculation of the skin in absolute units (21, 22). Microcirculatory function measures peak oxygen saturation after arterial occlusion. The post-occlusive reactive hyperemia phase refers to the increase in blood flow that mirror the endothelial vasodilatation function. The method is new but validated (23) and can detect disturbed microcirculatory flow in the skin not previously described for this patient group.

We observed that patients with SLE had impaired microcirculation as reflected by OxyP, compared to healthy controls. In healthy controls, the OxyP value were relatively stable regardless of age, whereas OxyP tended to decrease with age in patients with SLE. Stücker et al., using high-resolution two-dimensional laser Doppler perfusion imaging in a healthy group could not find age-related differences in microcirculatory perfusion (36).

Jonasson et al. demonstrated that microcirculatory perfusion is reduced in diabetic patients independent of microvascular changes in the kidneys and large-vessel stiffness (24). In the large population-based Swedish study SCAPIS, Jonasson et al. have shown that age and sex are important variables to consider in studies of microvascular function. They further analyzed groups with diabetes, hypertension and dyslipedemia and found that all these groups had lower OxyP levels compared to subjects without these diseases. The diabetic patients had the lowest values, which is in line with SLE patients in our study. The patients with hypertension or dyslipidemia showed slightly higher levels, but still decreased compared to patient without these diseases. They also observed that women had higher OxyP compared to men and that age influenced the value (25). Herein, we observed impaired OxyP also in patients of younger age.

Our study showed that reduced OxyP was negatively correlated with waist circumference, sagittal abdominal diameter, presence of Raynaud's phenomenon, as well as ARB/ACE inhibitor and statin therapy in the univariate analysis. In the multivariate analysis only presence of Raynaud's and use of statins remained as negatively correlating factors. However, it should be noted that statin therapy in the study population was prescribed as either primary or secondary prophylaxis. Mosdósi et al. investigated skin perfusion in finger with laser Doppler technology in adolescents with Raynaud's and reported altered heat-induced cutaneous hyperaemia responses (37). Thus, microcirculation is impaired in patients with Raynaud's, also when examined on the upper arm.

Statin therapy has been reported to impair hyperemic blood flow measured with laser Doppler (38). One explanation of our results may be that statin therapy represents dyslipidemia possibly not treated in all aspects. Dyslipdemia in SLE is often characterized by hypertriglyceridemia and decreased levels of high-density lipoprotein cholesterol (39). In active SLE, the atherogenic lipoprotein profile may result in accumulation of triglyceride-rich proteins and development of small and dense low-density lipoprotein-cholesterol particles (40–42).

The possible mechanism of microvascular abnormalities in SLE include autoantibodies forming immune complexes that deposit in small vessels and activate endothelial cells (18). However, SLE-associated risk factors such as disease duration, acquired organ damage (SDI), disease activity (SLEDAI-2K), C3, C4 or anti-dsDNA levels did not correlate with OxyP.

Increased AIx75 was observed in the SLE group compared to controls. Belizna et al. reported that patients with APS, both primary and secondary had a significantly higher prevalence of increased IMT, arterial stiffness, and presence of plaques, independent of known cardiovascular risk factors, compared with controls (43). Possibly, due to the low number of APS patients (*n* = 20), our study could not detect any association of APS and these parameters. Brodszki et al. demonstrated that vascular stiffness was associated with SLE irrespective of secondary vasculitis or not. They suggested that other mechanisms such as shifts in the collagen/elastin ratios, besides atherosclerosis might be involved in the pathogenesis of arterial stiffness in SLE (44). Both these studies used US to evaluate stiffness in large arteries.

Stortz et al. used evaluation of carotid-femoral PWV in patients with SLE, and showed an independent correlation between eGFR and PWV in SLE patients, and they found no difference between SLE and healthy controls (45). Another study using PWV demonstrated significantly higher aortic stiffness in patients with mixed connective tissue disease (MCTD) (46). In the present study, we used pulse wave analysis with tonometry of the radial artery, which is a muscular artery. This method can be used as a surrogate measure for arterial stiffness and cardiovascular risk (13). PWA is widely used to evaluate vascular properties, as it reflects the condition of the entire arterial system. The method is valuable, validated, and used for monitoring of hypertension (15). In a large population study on patients with low risk of CVD, Janner et al. showed that AIx reaches a plateau after 60 years of age, suggesting that AIx may be a better marker of CVD risk in younger subjects (47).

However, AIx is altered by changes in peripheral vascular tone and arterial stiffness, and measurement of PWV is needed for direct assessment of arterial stiffness.

A previous study, which included post-menopausal women with SLE, observed higher aortic PWV while central blood pressure and AIx were essentially unaltered (16). This may imply that PWV is a more sensitive tool for revealing early alteration in arterial wall geometry and function in subjects with SLE, while the later stiffening of central elastic arteries also shifts the reflection sites more distally. It is thus plausible that patients with SLE reaches their AIx plateau at an earlier age while controls still increase their augmentation to catch up the gap at higher age. Shang et al. used AIx to measure arterial stiffness and found an association with global disease activity assessed by SLEDAI-2K (48).

Increased arterial stiffness, including increased AIx, has been reported with increasing age, hypertension, hypercholesterolemia, end stage renal disease, and diabetes (49). However, gender is also a factor to include (50). When all significant variables were included in a multiple linear regression model in our study, age, female sex, and diastolic blood pressure remained as significant risk factors for AIx75. For younger women arterial stiffness is lower than in aged-matched men, but this sex difference reverses during aging (51). We only had eight men in the study and the age profile did not differ compared to the women. AIx75 also correlated with IMT in multiple vascular areas.

In our study, total cholesterol and triglycerides showed association with AIx75 in the univariate, but not in the multivariate, analyses. In contrast to healthy controls, vascular stiffness was improved (lower AIx75) by physical activity in univariate analysis. This finding should be further investigated.

Peak oxygen saturation correlated inversely with arterial stiffness (AIx75) implying that both methods could add valuable information regarding vascular status in SLE.

Although our study population was well-characterized, the number of included subjects remained limited. Nevertheless, the included number of patients were comparable with many other studies investigating the increased risk of cardiovascular disease in SLE (6, 8, 16, 17, 19, 43, 44–46, 48). We admit that both BMI and smoking habits were different among SLE patients compared to the controls. It cannot be excluded that these differences might have affected the results over all. The present study was based on the identical study population as our previous study (8), which could be considered as a limitation. No specific interventions were taken to avoid potential bias. However, the topic herein was different (with focus on microcirculation and vascular hemodynamics) and the collected data thus respond to separate research questions.

Ethnicity of the study population constitutes another limitation. Mainly Caucasian individuals were enrolled, and as ethnicity is well known to affect SLE severity, extrapolation of our results to other populations should be done with caution. Evaluation of microcirculation could be difficult to compare with established methods as the way of analyzing is completely new. However, our method has been validated for various diseases. Test-retest variability was not possible to study for the microcirculation method as the hyperaemic phase could affect a second measure. The described vascular methods are to some degree operator dependent. However, as only one operator was involved, at least the potential inter-operator dependent affection on the results was eliminated.

## Conclusion

In conclusion, our data suggest that adding non-invasive measurements of microcirculation and pulse wave analysis to standardized US of multiple arterial areas could be useful in the detection of vascular status in SLE. Impaired microcirculation as reflected by OxyP, and higher AIx were observed in SLE but not among controls, and both methods correlated with IMT and occurrence of plaque. The novel method with measurement of OxyP has not previously been studied in this group of patients but our results demonstrate that SLE patients have impaired microcirculation even in the younger ages. Further studies of microcirculation related to vascular hemodynamics in comparison to established methods for evaluation of cardiovascular risk in SLE are warranted.

## Data Availability Statement

The original contributions presented in the study are included in the article/ Supplementary Material, further inquiries can be directed to the corresponding author/s.

## Ethics Statement

The studies involving human participants were reviewed and approved by the Regional Ethics Board in Linköping (ref. M75-08, 2013/33-31 and ref. 2017/572-32). The patients/participants provided their written informed consent to participate in this study.

## Author Contributions

CSv, PE, CSj, and HZ contributed to the conception of this study and the study design. CSv collected and assembled data and wrote the original draft. CSv, PE, CSj, HZ, HJ, TS, and NB contributed to the analysis and interpretation of the data. All authors contributed to the critical revision of the article for important intellectual content and gave final approval of the article for submission. All authors have seen and approved the final text.

## Funding

The study was supported by Region Östergötland (ALF grants), the Swedish Rheumatism Association, the King Gustaf V's 80-year Anniversary Foundation, the King Gustaf V and Queen Victoria's Freemasons Foundation, the Gustafsson Foundation, and Linköping University Hospital Research Funds.

## Conflict of Interest

The authors declare that the research was conducted in the absence of any commercial or financial relationships that could be construed as a potential conflict of interest.

## Publisher's Note

All claims expressed in this article are solely those of the authors and do not necessarily represent those of their affiliated organizations, or those of the publisher, the editors and the reviewers. Any product that may be evaluated in this article, or claim that may be made by its manufacturer, is not guaranteed or endorsed by the publisher.

## Abbreviations

ACE: Angiotensin-converting enzyme
ACR: American college of rheumatology
AIx: Augmentation Index
AIx75: Augmentation Index normalized to heart rate 75
APS: Antiphospholipid syndrome
ARB: Angiotensin II receptor blocker
AXA: Axillar artery
CCA: Common carotid artery
CFA: Common femoral artery
DRS: Diffuse reflectance spectroscopy
hsCRP: High sensitive C-reactive protein
CVD: Cardiovascular disease
HDL: High-density lipoprotein
ICA: Internal carotid artery
IMT: Intima-media thickness
LDF: Laser Doppler flowmetry
LDL: Low-density lipoprotein
LN: Lupus Nephritis
MCTD: Mixed connective tissue disease
OxyP: Peak oxygen saturation
PWA: Pulse wave analysis
PWV: Pulse wave velocity
PORH: Post occlusive reactive hyperemia
SCA: Subclavian artery
SCAPIS: Swedish Cardiopulmonary BioImage Study
SFA: Superficial femoral artery
SDI: SLICC Damage Index
SLICC: Systemic Lupus International Collaborating Clinics
SLE: Systemic lupus erythemathosus
SLEDAI: SLE disease activity index
US: Ultrasound.
